# Risk Factors for Chronic Non-Communicable Diseases and Osteoporotic Fractures in a Middle and Elderly-Aged Population

**DOI:** 10.3390/jpm12091475

**Published:** 2022-09-09

**Authors:** Elena Mazurenko, Oksana Rymar, Victor Rerikh, Yuliya Khrapova, Artem Direev, Liliya Shcherbakova, Sofia Malyutina

**Affiliations:** 1 Research Institute of Internal and Preventive Medicine—Branch of the Institute of Cytology and Genetics, Siberian Branch of Russian Academy of Sciences, 630089 Novosibirsk, Russia; 2 Novosibirsk Research Institute of Traumatology and Orthopedics Named after Ya. L. Tsivyan, 630112 Novosibirsk, Russia

**Keywords:** chronic non-communicable diseases, lipid metabolism, osteoporosis, osteoporotic fracture, risk factors

## Abstract

**Aim**. To study the associations of risk factors for chronic non-communicable diseases (CNCDs) and osteoporotic fractures (OFs) in a population sampling over 50 years. **Materials and Methods**. The data of a cross-sectional population-based study obtained in the Russian part of the international project HAPIEE (Novosibirsk) are analyzed. The present analysis comprised 7363 men and women aged 50–69 years old. We have assessed the frequency of OFs for the last 12 months and risk factors of CNCDs. Cross-sectional associations between OF history and potential determinants were analyzed using multivariable-adjusted logistic regression. **Results**. The frequency of OFs in the last 12 months was 3.6% (3.2% in men and 4.0% in women, *p* = 0.074). In men, the probability of OFs increased with an elevation of blood pressure (BP), high-density lipoprotein cholesterol (HDL-C), ethanol consumption, and reduced with increased body mass index (BMI). In women, the probability of a fracture increased with current smoking and an increased duration of post-menopause and reduced with an increase in triglycerides (TG) levels, independently of other factors. **Conclusions**. A syndemia of risk factors, both generally recognized for OFs (BMI, tobacco smoking, alcohol consumption, postmenopausal duration) and new factors associated with CNCDs (BP, HDL, TG), have been defined.

## 1. Introduction

Although longevity is a global progress, the problem of the accumulation of CNCDs in advanced age is becoming increasingly relevant. According to WHO experts, osteoporosis is reaching epidemic proportions. Thus, along with other CNCDs, it should be classified as a socially significant health problem in many countries, including the Russian Federation [1]. Every year, over 8.9 million OFs occur worldwide [2]. There are 14 million people with osteoporosis in the Russian Federation. A total of 20 million have osteopenia and a high risk of OFs [1]. According to population research data, a high OFs frequency of the distal forearm (DF), 3.9%, was also revealed in Novosibirsk in patients aged 55–84 years [3].

Substantial evidence relates CNCDs and osteoporosis. However, the basic mechanisms of their pathogenesis in the elderly population remain controversial. It has been established that cardiovascular diseases (CVDs) are associated with a high risk of hip fractures [4]. Similarly, it has been shown that low bone mass in women may be an independent predictor of CVDs [5]. Unfavorable lipid profiles are the primary causes of CVDs and can affect bone health and the risk of OFs through many mechanisms, including oxidative stress, enhanced systemic inflammation, decreased blood supply to bones, and changes in bone remodeling [6]. The record shows that type 2 diabetes mellitus (T2DM) is also a risk factor for hip fracture. Recent studies have shown that microstructural changes in bone tissue are more pronounced among people with T2DM and microvascular complications [7,8,9]. In particular, according to our data, the comorbidity of T2DM, complicated by diabetic retinopathy with musculoskeletal disorders in the elderly population, was detected [10].

In this regard, it is relevant to study the association of risk factors for CNCDs, such as obesity, dyslipidemia, hyperglycemia, tobacco smoking, alcohol consumption, and hypertension (HT) with the development of OFs. No studies on this issue have been conducted at the population level in Russia.

The aim of the study was to assess the associations of risk factors for CNCDs and OFs in a population sampling over 50 years (Novosibirsk).

## 2. Materials and Methods

### 2.1. Participants

The study was performed on the material of a population cohort examined in Novosibirsk (the Russian arm of the multicenter project “Determinants of cardiovascular dis-eases in Central and Eastern Europe: cohort study”, the HAPIEE project). A random sample of men and women aged 45–69 was formed among residents of two districts, typical for Novosibirsk in terms of infrastructure, demographic indicators and the level of population migration. The sample was formed on the basis of electoral lists using a table of random numbers and stratified by 5-year age groups. The design and protocol of the project have been published previously [11]. At baseline, 9360 subjects were examined in 2003–2005 (men—45.6%, women—54.9%; 98% Caucasians, response rate—61%). The cohort was re-examined twice in 2006/08 and 2015/18. The sample size and age structure were defined by the protocol of the HAPIEE international project [11].

Participants were invited to participate in the study by three letters. The study was performed at the survey center of the Research Institute of Internal and Preventive Medicine: Branch of the Institute of Cytology and Genetics, Siberian Branch of the Russian Academy of Sciences. The data were collected using standardized and structured questionnaires and measurements with the assistance of trained and standardized medical staff (http://www.ucl.ac.uk/easteurope/hapiee-cohort.htm accessed on 6 September 2022).

The present analysis included men and women aged 50–69 years (*n* = 7363); only postmenopausal women were included (average duration 12.2 ± 7.2 years). Those who did not have the blood chemistry test performed (*n* = 156), persons younger than 50 (*n* = 1581), and women in the reproductive period (*n* = 260) were excluded from the study. The present paper’s design is a cross-sectional study.

### 2.2. Study Questionnaire

Using structured questionnaires of the HAPIEE project (http://www.ucl.ac.uk/easteurope/hapiee-cohort.htm), (accessed on 6 September 2022), socio-demographic variables were evaluated for all persons included in the study. At the time of the study, information was collected on the prevalence of fractures over the past 12 months. The medical history and treatment of T2DM, the main risk factors for CNCDs (obesity (BMI ≥ 30 kg/m^2^), hyperglycemia, dyslipidemia, duration of menopause in women, smoking, alcohol consumption), and hormone replacement therapy in postmenopausal women were also evaluated.

Anthropometric measurements were used to assess height, weight, and waist circumference (WC) with the calculation of BMI. Height and body weight were measured in the standing position, without outerwear and shoes. For these purposes, a standard stadiometer with an accuracy of 0.5 cm and mechanical scales that passed metrological control (the measurement accuracy—0.1 kg) were used. The body mass index was calculated by the formula:

BMI (kg/m^2^) = body weight (kg)/height^2^ (m^2^)

Blood pressure was measured three times by OMRON M5-I on the right hand in a sitting position after a five-minute rest with 2 min intervals between measurements. The average value of threefold blood pressure measurements was calculated. Hypertension was diagnosed at levels of SBP ≥ 140 mm Hg or DBP ≥ 90 mm Hg (ESC, 2018) and in individuals with normal blood pressure readings while taking antihypertensive drugs during the last two weeks before the examination.

The smoking questionnaire included information on the current smoking of one or more cigarettes per day and smoking in the past. A person who smokes at least one cigarette a day was considered a smoker.

Alcohol consumption was evaluated using the Graduated Frequency Questionnaire (GFR) [12] and alcohol intake was converted to pure ethanol. According to the GFR data, values exceeding the WHO safe alcohol consumption threshold were calculated (more than 30 g of pure ethanol for men and 20 g for women, as the average alcohol dosage for a session).

A low-energy fracture was established according to the anamnesis, if it occurred when falling from a person’s height, or spontaneously in the presence of radiographic verification.

T2DM was established according to epidemiological criteria such as fasting plasma glucose levels (FPG) = 7.0 mmol/l [13] and/or normoglycemia in persons with a history of established T2DM and the fact of treatment.

The antihypertensive therapy received was assessed by the regular intake of medications during the last two weeks. The drug dosage was ignored.

### 2.3. Biochemical Measurements

Blood biochemistry tests were drawn in a sitting position, on an empty stomach, from the ulnar vein with a vacutainer. After centrifugation, the blood serum was stored in a low-temperature chamber (−70 °C). A blood biochemistry test was performed in the Laboratory of Clinical Biochemistry of the Research Institute of Internal and Preventive Medicine—Branch of the Institute of Cytology and Genetics, Siberian Branch of the Russian Academy of Sciences, standardizing internal and external federal quality control. Determination of the total cholesterol (TC), HDL-C, and TG levels were conducted by the enzymatic method using Biocon commercial standard kits (Monchberg, Germany) on the KoneLab autoanalyzer (Waltham, MA, USA). The concentration of low-density lipoprotein cholesterol (LDL-C) was calculated using the W.T. Friedewald formula:

LDL-C = TC − (TG/2.2 + HDL-C) (mg/dL)

A preliminary calculation of the sample size for this analysis (in each group) was performed to ensure a minimum odds ratio OR = 2 (when a = 0.05; b = 0.10 and 0.20) for the prevalence of the tested factor of 10% and 20% in the control.

### 2.4. Statistical Analysis

Statistical data processing was conducted using the SPSS software package (v.13.0, New York, NY, USA). The statistical significance of the differences in average indicators was evaluated by the Student’s *t*-test for normally distributed characteristics in the presence of two groups; if more than two groups with normal distribution were analyzed, ANOVA with Bonferroni correction was used. The comparison of two independent groups by quantitative characteristics with a non-normal distribution was made using the non-parametric Mann–Whitney U-test. We used Pearson’s chi-squared test (χ^2^) to determine the statistical significance of the differences in qualitative characteristics. The data obtained in the tables and text are shown as absolute and relative values (n, %) and also as (M ± σ), where M—arithmetic mean, σ—standard deviation. The differences were considered statistically significant at *p* < 0.05.

To assess the relationship between risk factors and their combinations with OFs, logistic regression was used (unstandardized, age-standardized, and multivariate models were analyzed). The models used continuous variables (age, BMI, TG, HDL-C, SBP, DBP) and categorical variables (presence of T2DM versus (vs.) individuals without T2DM; alcohol consumption ≥ 30 g and 20 g of pure ethanol per session for men (m) and women (w), respectively, vs. alcohol consumption < 30 g (m) and <20 g (w) of ethanol per session; current tobacco smoking and in the past vs. non-smokers). The relative risk of fractures was evaluated using the odds ratio (OR) indicator.

## 3. Results

Among the 7363 individuals included in the analysis from the basic screening, the number of men was 3511 (average age 60.3 ± 5.8 years) and women was 3852 (average age 60.8 ± 5.6 years); 3.6% (267 people) indicated OFs that occurred during the last 12 months: 3.2% of men and 4.0% of women, *p* = 0.074. Table 1 and Table 2 show the basic clinical and laboratory features of men and women in the groups who suffered an osteoporotic fracture in the last 12 months at the time of the study (2003–2005) and in groups without a history of fractures.

Men with fractures had lower values of weight and BMI (*p* = 0.038, *p* = 0.030) and higher levels of HDL-C and DBP; they also consumed alcohol more often at a dose of more than 30 g of pure ethanol per session (*p* = 0.001, *p* = 0.050, *p* = 0.002, respectively) than men without fractures. Women with OFs had significantly lowed mean TG values than women without fractures (*p* = 0.044).

**Figure 1:**
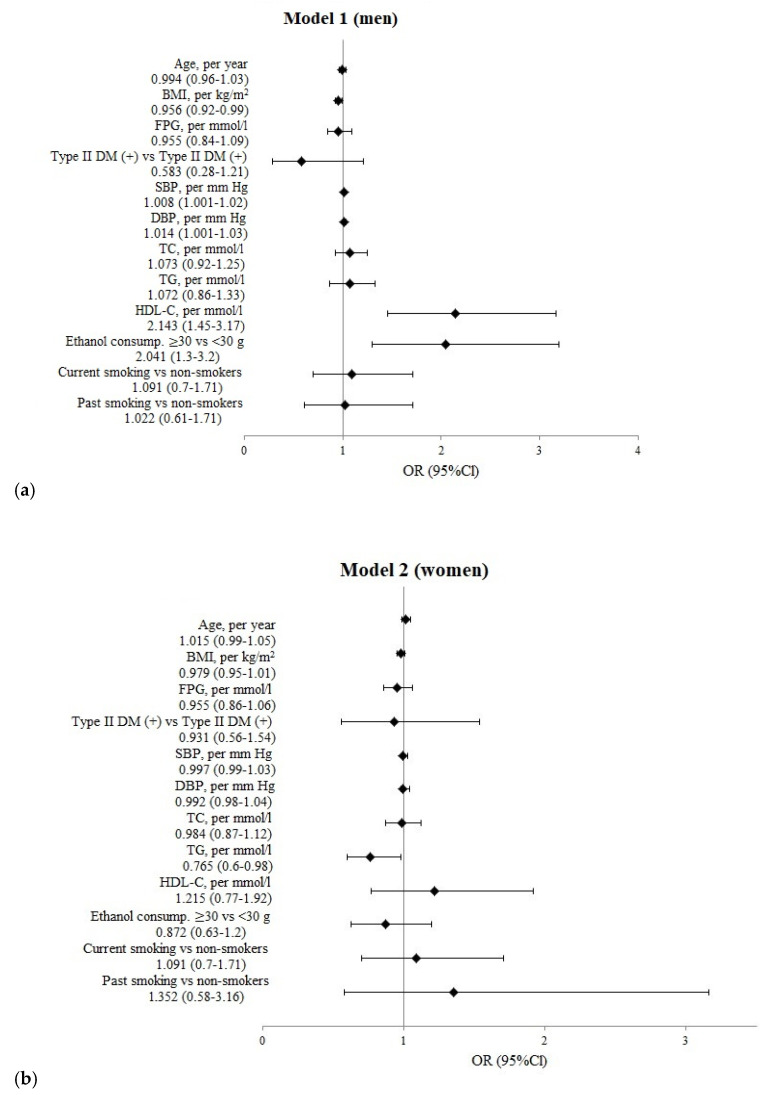
The logistic regression analysis results of the association of risk factors of CNCDs with the risk of OFs over the past 12 months in individuals aged 50–69 years in age-standardized models. (**a**) **Model 1** for men, (**b**) **Model 2** for women.

When performing a logistic regression analysis of risk factors in age-standardized models (Figure 1), the risk of OFs in men grew with increased blood pressure, HDL-C, and ethanol consumption of more than 30 g per session and reduced with an increase in BMI. The risk of OFs in women decreased in 12 months with an increased TG level.

**Figure 2:**
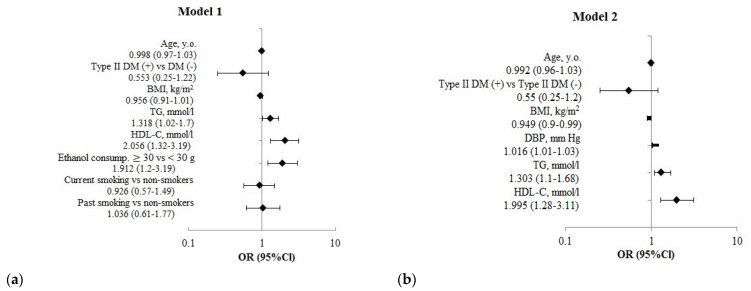
The multivariate logistic regression analysis results of the association of risk factors of CNCDs with the risk of fractures over the past 12 months in men aged 50–69 years (*n* = 3511). (**a**) **Model 1**—adjusted for age, T2DM, BMI, TG, smoking, ethanol consumption. (**b**) **Model 2**—adjusted for age, T2DM, DBP, TG, HDL-C.

Among men in multivariate models (Figure 2), the revealed risk associations of OFs were similar to the results obtained in the univariate analysis.

**Figure 3:**
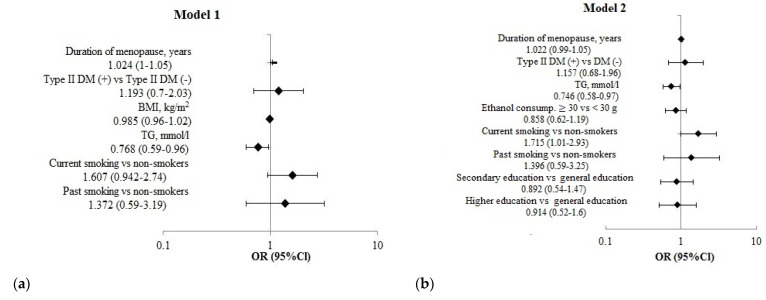
The multivariate logistic regression analysis results of the association of risk factors of CNCDs with the risk of fractures over the past 12 months in women aged 50–69 years (*n* = 3852). (**a**) **Model 1**—adjusted for T2DM, BMI, TG, smoking, duration of menopause. (**b**) **Model 2**—adjusted for T2DM, TG, ethanol consumption, smoking, education, duration of menopause.

The fracture risk rose in women with current smoking and increased postmenopausal duration. The fracture risk decreased with increasing TG, regardless of other factors (Figure 3).

## 4. Discussion

There are different incidence rates of OFs worldwide. According to 2010 data, 3.5 million low-energy fractures were recorded in the European Union [14]. In the Russian Federation, over a 2-year period, 1265.0 OFs were registered in Pervouralsk per 100,000 residents aged 50 and over (1477.1 among women and 923.1 among men) [15]. According to our study, in the last 12 months, 3.6% of people aged 50–69 also had a high incidence of OFs.

It is believed that, epidemiologically, osteoporosis is associated with atherosclerosis and hyperlipidemia. In our study, we obtained a favorable association of fractures with an increase in HDL-C and TG in men and an unfavorable association with an increase in TG in women. Similar outcomes are shown in a meta-analysis by Y.-Y. Chen et al. The authors showed that serum levels of HDL-C are higher in women with postmenopausal osteoporosis than in the group with normal bone density. Meanwhile, TG levels were lower in the osteoporosis group, but they did not reach a statistical difference [16]. A meta-analysis of large observational studies performed by S. Ghorabi et al., 2019 [17] revealed significant favorable associations in one of seven studies between TG levels and OFs. Currently, it is complicated to explain the obtained sexual dimorphism and the relationship of OFs with the TG level. Nevertheless, different associations between lipid metabolism and OFs are found in large studies, not only depending on gender but also depending on age. In a study by Chen X. et al. 2016, the probability of osteoporosis grew with an increased HDL in persons under 65 and reduced with an increase in TG in persons over 65. Perhaps these data indicate that the associations obtained, both in our study and in other large ones, may fluctuate in different age groups [18].

Studies that have evaluated the relationship between HDL-C and bone mineral density (BMD) have shown different results. Some papers have demonstrated a negative correlation [19]. In a population-based study in Tromso, in which 27,159 people were observed for six years, the protective effect of lower HDL-C values on vertebral fractures was proven [20].

We expected HDL to have a positive effect on bone health due to its anti-inflammatory properties. However, adipocytes and osteoblasts have a common precursor—The mesenchymal stem cell (MSC) [21]. It has been shown that an increase in marrow adipose tissue is associated with bone loss [22]. Kha et al. reported that osteogenic differentiation of MSCs may be stimulated by specific oxysterols. Consequently, a high level of HDL, which can remove oxysterols from peripheral tissues, has a negative effect on osteogenic differentiation [21,23].

It has been established that alcohol harms calcium metabolism and other metabolic processes. The meta-analysis [24] suggested that alcohol consumption daily at a dose of 2 units or less does not significantly increase the risk of fractures, but an increased risk of any fracture was registered above this dose. In our study, the risk of fractures increases by 1.9 times with ethanol consumption of more than 30 g per intake in men but not in women. A possible reason for sexual dimorphism may be that men are more likely to have worse behavior when drinking alcohol and more severe episodes of alcohol consumption [25].

In our study, smoking increased the risk of fractures in women by 1.7 times, regardless of other factors, but not in men. J.A. Kanis [26] reported in the meta-analysis that smoking is associated with a significantly increased fracture risk, both in women and men. The revealed association of smoking with fractures only in women in our study is probably because all the women were going through the menopause and as is known, the menopause occurs earlier in women than andropause does in men.

Epidemiological and biological studies concerning the pathogenesis of OFs and HT have proved the hypothesis that diseases have several similar etiopathogenesis components [27]. In our study, a positive association between HT and OFs was detected. Such a result is consistent with studies by S. Yazici et al. (2011), where HT acts as an independent predictor of spinal osteopenia and OFs [28].

According to our study, the fracture risk decreases with increased BMI. It is traditionally considered that increased body weight has a protective effect on the bone, which is reflected in our study. This is due to increased mechanical stress on the skeleton and the ability of adipocytes to convert androgens into 17β-estradiol, which increases MSC [29]. There are other favorable factors associated with bone strength in obese people. The great thickness of soft tissues on the lateral area of the thigh mitigates falls and thus can act as a protective factor against hip fracture with a high body weight, even if the load to force ratio is exceeded.

The study has limitations. Assessment of the history of OFs was performed using a standardized questionnaire without X-ray control. In this regard, asymptomatic compression fractures of the vertebral bodies could not be diagnosed. However, the advantage of this study is a large volume of random population sampling (*n* = 7363). It is also worth noting that the survey on the presence of risk factors and OFs was performed by an endocrinologist, which made it possible to most accurately gather medical history and minimize errors in identifying the studied pathology.

Despite the fact that the study was conducted in 2003–2005, the data obtained as a result of this study help to understand the deeper links between CNCDs and OFs. This study is the first cross-sectional study to explore the association between risk factors and OF fracture in the largest available sample. At the same time, this study creates a basis for the further study of risk factors for the long-term risk of fractures, validation of the FRAX scale in the Siberian region and, possibly, the creation of the Siberian risk scale for osteoporotic fractures. Given the lack of population-based studies exploring the risk factors for chronic CNCDs and OFs in Siberia, these data are relevant, given the more severe weather conditions in Siberia, and are practically significant for diagnosing and assessing the risk of fractures in Novosibirsk residents. To date, the results of this study have been used as the basis for a series of subsequent analyses in the context of this topic.

## 5. Conclusions

Therefore, a high incidence of OFs over the past 12 months was detected in individuals aged 50–69 years, amounting to 3.6%. The syndemia of risk factors, both generally recognized for OFs (BMI, tobacco smoking, alcohol consumption, postmenopausal duration) and new factors associated with CNCDs (SBP, DBP, HDL, TG), has been defined, implying significant links in the development of CNCDs and OFs.

## Figures and Tables

**Table 1:** Risk factors for CNCDs in men who have suffered fractures in the last 12 months and without fractures in history (2003–2005).

Indicators	Fractures+	Fractures−	*p* ^1^
	(*n* = 113)	(*n* = 3398)	
Age, y.o.	60.1 ± 5.6	60.3 ± 5.8	*0.689*
Height, cm	170.4 ± 5.6	170.7 ± 6.3	*0.623*
Weight, kg	75.0 ± 13.4	77.9 ± 14.4	***0.038***
BMI, kg/m^2^	25.8 ± 4.0	26.7 ± 4.4	***0.030***
BMI ≥ 30 kg/m^2^, (n/%)	20/17.7%	725/21.3%	*0.352*
WC, cm	92.2 ± 12.3	94.5 ± 12.2	*0.052*
SBP, mm Hg	150.0 ± 25.4	145.9 ± 23.7	*0.110*
DBP, mm Hg	93.6 ± 15.0	91.1 ± 13.4	***0.050***
FPG, mmol/L	5.9 ± 1.8	6.1 ± 1.7	*0.477*
TC, mmol/L	6.1 ± 1.3	6.0 ± 1.2	*0.713*
TG, mmol/L	1.5 ± 0.9	1.5 ± 0.8	*0.904*
HDL-C, mmol/L	1.6 ± 0.5	1.5 ± 0.4	***0.001***
LDL-C, mmol/L	3.7 ± 1.1	3.8 ± 1.1	*0.317*
Tobacco smoking, (n/%)			
Non-smokers	28/24.8%	909/26.8%	*0.641*
Past smokers	28/24.8%	896/26.4%	*0.706*
Current smokers	57/50.4%	1593/46.9%	*0.456*
Alcohol consumption: average ethanol consumption (g)			
≥30 g per session, (n/%)	89/78.8%	2190/64.4%	***0.002***
≥30 g per day, (n/%)	16/14.2%	400/11.8%	*0.441*
Education (n/%)			
Basic general education	9/8.0%	442/13.0%	*0.115*
Secondary (complete) education	72/63.7%	1862/54.8%	*0.061*
Higher education	32/28.3%	1094/32.2%	*0.385*
T2DM (n/%)	8/7.1%	394/11.6%	*0.138*
HT, (n/%)	78/69.6%	2279/67.1%	*0.663*

^1^ The values are presented as M ± σ or n/%; «Fractures+»—individuals who have had fractures in the last 12 months at the time of the study, «Fractures−»—individuals who have not had fractures in the last 12 months at the time of the study.

**Table 2:** Risk factors for CNCDs in women who have suffered fractures in the last 12 months and without a history of fractures (2003–2005).

Indicators	Fractures+	Fractures−	*p* ^1^
	(*n* = 154)	(*n* = 3704)	
Age, y.o.	61.2 ± 5.9	60.8 ± 5.6	*0.331*
Height, cm	158.1 ± 6.5	157.4 ± 5.8	*0.165*
Weight, kg	74.4 ± 13.5	75.5 ± 14.6	*0.332*
BMI, kg/m^2^	29.8 ± 5.2	30.5 ± 5.6	*0.123*
BMI ≥ 30 kg/m^2^, (n/%)	72/46.8%	1812/49.0%	*0.585*
WC, cm	91.9 ± 12.4	92.9 ± 13.2	*0.343*
SBP, mm Hg	146.7 ± 27.9	148.2 ± 26.3	*0.494*
DBP, mm Hg	90.0 ± 14.5	91.4 ± 13.4	*0.213*
FPG, mmol/L	6.0 ± 1.4	6.1 ± 1.8	*0.436*
TC, mmol/L	6.6 ± 1.3	6.7 ± 1.3	*0.911*
TG, mmol/L	1.5 ± 0.6	1.6 ± 0.9	***0.044***
HDL-C, mmol/L	1.6 ± 0.4	1.6 ± 0.3	*0.400*
LDL-C, mmol/L	4.4 ± 1.2	4.3 ± 1.2	*0.752*
Tobacco smoking, (n/%)			
Non-smokers	131/85.1%	3289/88.9%	*0.136*
Past smokers	6/3.9	121/3.3%	*0.670*
Current smokers	17/11.0%	288/7.8%	*0.143*
Alcohol consumption: average ethanol consumption (g)			
≥ 20 g per session, (n/%)	72/46.8%	1855/50.2%	*0.407*
≥ 20 g per day, (n/%)	1/0.6%	14/0.4%	*0.597*
Education, (n/%)			
Basic general education	21/13.6%	442/12.0%	*0.528*
Secondary (complete) education	95/61.7%	2343/63.4%	*0.674*
Higher education	38/24.7%	913/24.7%	*0.997*
Menopause duration, years	13.2 ± 7.6	12.2 ± 7.2	*0.093*
Receive HRT, (n/%)	9/5.8%	251/6.8%	*0.647*
T2DM (n/%)	18/11.7%	451/12.2%	*0.850*
HT, (n/%)	108/70.1%	2705/73.1%	*0.408*

^1^ The values are presented as M ± σ or n/% «Fractures+»—individuals who have suffered fractures in the last 12 months at the time of the study, «Fractures−»—individuals who have not had fractures in the last 12 months at the time of the study.

## Data Availability

The data presented in this study are available in tabulated form on request. The data are not publicly available due to ethical restrictions and project regulations.
